# An interpretive descriptive approach of patients with osteoporosis and integrating osteoporosis management advice into their lifestyle

**DOI:** 10.1080/17482631.2022.2070976

**Published:** 2022-05-01

**Authors:** Christina Ziebart, Joy MacDermid, Rochelle Furtado, Tatiana Pontes, Mike Szekeres, Nina Suh, Aliya Khan

**Affiliations:** a Department of Rehabilitation Sciences, Faculty of Health Science, Western University, London, Ontario, Canada; b Physical Therapy and Surgery, Western University, London, Ontario, Canada; c Roth McFarlane Hand and Upper Limb Centre, St. Joseph’s Hospital, London, Ontario, Canada; d Occupational Therapy, Boston University Academy, boston, Massachusetts, USA; e Department of Orthopaedics, Emory University, Atlanta, Georgia, USA; f McMaster University, Ontario, Canada

**Keywords:** Osteoporosis, nutrition, exercise, falls prevention, qualitative

## Abstract

**Introduction:**

Although osteoporosis-exercise recommendations have been established, implementation of the information remains a challenge for people with osteoporosis. This study aimed to understand how participants integrate osteoporosis management advice into their lifestyle and the challenges they might face.

**Methods:**

Integrative descriptive methods were used for this qualitative study. In-depth interviews were conducted with 13 Canadian participants (age range 51–90) that knew they had osteoporosis. Participants were asked to participate in one-on-one interviews; discussing exercise, nutrition and falls prevention for people with osteoporosis.

**Results:**

The following themes emerged from this study: understanding fragility fractures and fall risk, knowledge acquisition through personal and vicarious experience over the lifespan, awareness of environmental risks and opportunities, understanding the effect of exercise on the bones and in life, challenges managing exercise expectations, attitude towards non-pharmacological management.

**Conclusion:**

Participants recognized the benefit of non-pharmacological management for managing osteoporosis, but sometimes found it difficult to integrate into their daily activities due to lack of time or knowledge. Participants weren’t always clear on which component of their osteoporosis management should be prioritized.

## Introduction

Osteoporosis is a metabolic bone disease affecting one in three women and one in five men (Ensrud, 2013). Osteoporotic fractures account for 80% of all fractures in people over the age of 50, leading to increased morbidity and mortality (Ensrud, 2013). Osteoporotic fractures can occur from a low trauma incident (Prior et al., 2015), such as slipping on ice or tripping on a curb. With an ageing population the prevalence of osteoporotic fractures is expected to increase (Prior et al., 2015).

Clinical management of osteoporosis is approached through both pharmacological and non-pharmacological methods. Routine practice for a patient suspected of having osteoporosis consists of a physical examination by their physician to assess any height loss or changes in posture (Papaioannou et al., 2010). Height loss of greater than 2 cm in a year is an indication of a vertebral fracture (Genant et al., 2000; Ismail et al., 1999). Depending on the medical history, patients may be recommended to get a bone density scan (DXA), and then the clinician will determine the patient’s 10-year risk of fracture (Kanis, 1994). Patients are categorized into low, moderate, or high risk of fracture, and treatment strategies are based on fracture risk and disease progression, in conjunction with patient preferences and clinical expertise. Typically, those with low fracture risk are managed non-pharmacologically, while those at high risk require additional pharmacological management. Non-pharmacological management of osteoporosis encourages patients to exercise daily and have a diet with sufficient vitamin D and calcium-rich foods (Papaioannou et al., 2010).

Guidelines for exercise in people with osteoporosis have been established, with slight modifications for those that have sustained a vertebral fracture compared to those who have not (Giangregorio et al., 2014). Generally, the guidelines recommend 20-minutes of daily balance training, posture awareness, strength training each of the major muscle groups at least twice weekly and aerobic training, but not to the exclusion of strength training (Giangregorio et al., 2014). Despite clear pharmacological and non-pharmacological osteoporosis management guidelines, uptake and adherence to treatment continues to be a challenge.

Patients find it difficult to perceive and interpret the diagnosis of osteoporosis, their current risk of fracturing and knowing how to manage their day-to-day with osteoporosis (Hansen et al., 2014). Several studies have shown that the diagnosis of osteoporosis can lead to psychological and physical consequences, affecting quality of life (Nielsen et al., 2013; Reventlow, 2007; Reventlow & Bang, 2006; Weston et al., 2011; Wilkins, 2001). A systematic review of quantitative studies found that structure and psychological determinants of health behaviour need to be understood to better understand and manage osteoporosis(McLeod & Johnson, 2011). One strategy is through educational programmes, which may increase the patient’s knowledge of osteoporosis and improve their adherence to treatment but may not actually change health behaviour (Hjalmarson et al., 2007; Ryan, 2009; Tollén et al., 2011). Treatment for osteoporosis is multi-factorial. Currently there are studies available looking at how patients perceive pharmacological management, or non-pharmacological management, but no studies have looked at the patient’s perspective on both. It’s not clear if there are gaps in disseminating the information, and what information patients seem to prioritize. As well, many studies look at older adults with osteoporosis (over the age of 65; Gibbs et al., 2019; Hansen et al., 2014; McLeod & Johnson, 2011), despite evidence suggesting that people over the age of 50 should be screened for osteoporosis and could begin a non-pharmacological intervention(Compston et al., 2009; Kanis & McCloskey, 1998; Reid, 2020; Tella & Gallagher, 2014). There remain gaps in the literature in understanding patient’s perspective of how their osteoporosis has been managed non-pharmacologically.

This study aimed to understand how participants integrate osteoporosis management advice into their lifestyle and the challenges they might face.

## Methods

An interpretive descriptive methodology was used for this qualitative study (Hunt, 2009; Strauss & Corbin, 1994). Interpretive description is aligned with constructivist and naturalistic style of inquiry (Hunt, 2009; Thorne et al., 2004). This methodology is commonly used in a clinical context of applied health disciplines (Hunt, 2009; Thorne et al., 2004). The strength of interpretive descriptive methodologies is that there is a coherent logic and structure designed towards the generation of practice-relevant findings(Hunt, 2009; Thorne et al., 2004). However, there are also challenges in the degree of interpretation and that this is a lesser-known methodology (Hunt, 2009; Thorne et al., 2004). Generally, the methods for interpretive descriptive methodologies are similar to that of traditional qualitative methods but acknowledges that the researcher is involved in the study and may not be completely impartial (Thorne et al., 2004).

### Participants and procedures

In-depth interviews were conducted with 13 Canadian participants (age range 51–90) that knew they had osteoporosis or osteopenia, as told by their physician, taking place between February to April of 2019. Three participants refused to participate due to lack of interest in the project. Participants were asked to participate in a one-on-one interview. The interview guide was piloted with the first participant, but no major changes were made and therefore the data was included in the overall sample. Participants were encouraged to speak freely and openly about their experience with osteoporosis. The semi-structured interview guide was structured to prompt discussion around which medical professionals the participants have seen for their osteoporosis management, the advice they received about managing their osteoporosis, and a specific focus on whether they had received advice about exercise, nutrition, and falls prevention for osteoporosis. Goals and knowledge gaps related to osteoporosis management were explored to further understand how their osteoporosis care could be modified to better suit the requirements of living with osteoporosis.

Participants were selected from a medical clinic in Ontario, Canada. Eligible participants were selected based on having osteoporosis or osteopenia, being able to speak and understand English, and capable of providing informed consent to participate. Patients with secondary osteoporosis were excluded from this study. Participants were approached in the clinic and were provided with the letter of information and consent. Interested participants reached back out to the lead student researcher to set up an interview day and time. Participants had the opportunity to ask any questions about the letter of information before signing consent. The interview took place in a location of the participant’s preference, often a Starbucks. Participants were reminded that their conversation was taking place in a public location and to be mindful of that when answering questions. Participants signed the informed consent prior to being interviewed. The interviews were audio-recorded on a digital recorder. To achieve purposeful sampling, recruitment consisted of women in the earlier stages post-menopause, older women, and men. The sample provides both depth and different dimensions of emerging themes. The data were analysed throughout the data collection period to allow for constant comparison and an indication of when theoretical saturation was reached. Three authors (CZ, RF, and TP) performed the constant comparison and met frequently to discuss the findings. There were several instances where there was disagreement between themes, which were resolved by the supervising author. Constant comparison was used to assess the quality of the questions being asked, verifying that participants understood the questions, and allowing the evolution of questions. For example, it emerged that participants valued non-healthcare providers advice for their osteoporosis, so future iterations of the interview guide explored that theme more thoroughly. Data collection was ceased when theoretical saturation was reached, which is when no new information was provided in the last 2 interviews.

A total of 13 participants were interviewed. The average length of the interview was 45 minutes, with the shortest interview at 34 minutes and the longest interview at one hour and 15 minutes. The mean age was 66, mean age at menopause was 46, and mean number of fractures was two. A total of 12 females and one male was included, three participants have osteopenia and seven have osteoporosis, and two participants self-reported that they did not know (however the study physician did confirm they had osteoporosis). The fracture risk included participants in low, moderate, and high risk of fracture (Table I).

**Table I.** Participant demographics and response to demographic survey data

Age of participants (Mean (SD))	66 (10.9)
Age at menopause (Mean (SD))	46 (9.2)
Sex (N)	Female (2) Male (1)
Primary dwelling (N)	City (12) Rural (1)
Osteoporotic fracture (Mean (SD))	0.61 (0.87)
Location of fractures (N)	Wrist (5) Hand (1) Arm (2)
Diagnosis (N)	Osteoporosis (9) Osteopenia (3) Don’t know (1)
Self-Reported Fracture risk	Low (1) Moderate (3) High (4) Don’t know (5)
Taking osteoporotic medication	Yes (6) No (7)

### Data analysis

Data analysis occurred manually and simultaneously with the data collection (Gadamer et al., 2004; Strauss & Corbin, 1994). The interviews were stopped once theoretical saturation was reached. A single researcher (CZ) transcribed all the interviews verbatim. Three researchers (CZ, RF, and TP) analysed all transcripts independently and then discussed the codes to develop the themes. Data was coded sentence-by-sentence to identify emerging themes (Strauss & Corbin, 1994).

Data was first coded for major categories of information, where a category was defined as “concepts that pertain to the same phenomenon” (Strauss & Corbin, 1994). Relationship and similarities among categories were discussed leading to the formation of themes

Several strategies were employed to establish trustworthiness in this study (Shenton, 2004). The methods used in this study were based off previous similar studies (Hansen et al., 2014; Lowe et al., 2019), iterative questioning was used to ensure honest answers from participants, as well peer scrutiny was used in the final stages of the manuscript to reduce the risk of bias from the first author (Shenton, 2004). All these strategies were used to help improve the credibility of this work. Dependability was established by using an appropriate research design and implementation, and through reflective appraisal of the project (Shenton, 2004). As mentioned, three independent researchers analysed all the transcripts to reduce bias from the lead researcher. The codes were compared, and themes were established. The themes were then presented to a larger group of researchers, further supporting the trustworthiness of the data.

### Ethical considerations

This study was approved by the University’s Research Ethics Board and Lawson Health Research Board (#113,036). This project fulfils requirements on research including information, consent, confidentiality, and safety of the participants and was guided by the ethical principles: autonomy, beneficence, non-maleficence, and justice. Data collection was conducted with confidentiality and with informed consent from the participant. Participants received both verbal and written information about the project and that they could withdraw from the project at any time without any explanation.

## Results

The following themes emerged from this study: Understanding fragility fractures and fall risk, Knowledge acquisition through personal and vicarious experience over the lifespan, Awareness of environmental risks and opportunities, Understanding the effect of exercise on the bones and in life, Challenges managing exercise expectations, Attitude towards non-pharmacological management

### Understanding fragility fractures and fall risk

A recurring theme that arose from the interviews was understanding what was considered a fragility fracture, and which fractures might be considered osteoporotic fractures. Several of the participants mentioned that they broke a bone, which initiated the trajectory to getting diagnosed with osteoporosis. However, it was also common that the participants did not feel that bone frailty was the cause of the fracture. One participant explained that she tripped up a curb but felt it was high enough impact that she was not surprised she broke her wrist. There continues to be confusion around what is considered a low impact injury, what is a fragility fracture, and what the consequences of the fragility fracture are. For example, another participant mentioned that she knew she had a fragility fracture, but she did not believe that having a fragility fracture affected her future risk for fracture: “(…) I really don’t think so. I don’t want to say I don’t believe them (health professional). I just don’t think it’s as big of an issue as they first said.” Female, Age 70

Other participants relied upon imaging rather than health professional advice for their understanding of bone fragility: *“she said I was moderate to high risk for osteoporosis …… And then the fracture. And she showed me what a normal bone scan should be and what mine looked like.” Female, Age 60*

This type of conversation was common, as participants were unclear what their future risk of fracture was, however, there were times that they were aware of their risk of fracture but believed they could overcome it:

*“I think there are risks and life is full of risks, if a person becomes too obsessed with risk, (…) she lives a life that is a death. And I don’t want to do that. And I’m also not going to be stupid.” Female, Age 57*

However, the fear of falling made one of the interviewees stop performing activities and exercises due to the risk of fall and fracture:

“*I also said to myself you need to be more aware of how you walk. You have to stop doing things that could make you fall. But that’s when I stopped doing all the other physical stuff, the nice stuff that I did with the kids. Skating, tobogganing, I just stopped it all because I was worried about falling. And now, I have to tell you, because I even fell in my own kitchen*.” Female, Age 62

Although mentioned by the above interviewee, falling at home or work wasn’t a concern for all participants: “*I’m not really afraid of falling at home or work because I’m always holding onto the banisters. I won’t go up and down stairs without holding on*.” Female, Age 64

Fortunately, many of the participants recognized that exercise is helpful for reducing the risk of fracture and were able to correctly identify the benefits of exercise. One participant explained:

*“[exercise] sure helps in the risk factors if nothing else. If it is not going to build up or halt the progression of the degeneration, at least the strength you receive from the exercise certainly helps and the muscular strength helps as far as being high risk of fracture” – Female, Age 70*

Engaging in an exercise routine to reduce the risk of fracture was a motivator for the participants but understanding the impact of exercise on bone density was confusing.

### Knowledge acquisition through personal and vicarious experience over the lifespan

From the interviews, it was evident that participants drew a lot of their osteoporosis knowledge from past experiences or people that were close to them who had experienced osteoporosis. It was very common that participants drew on their parental experiences with osteoporosis to guide their management of osteoporosis:

*“ … because of my mom. She’s been to the falls prevention clinics over the years and obviously with [her husband] I knew right away and I was the one that said you have to have a walker now. And he didn’t want it at first, but of course he didn’t even want to have the cane. But now that he’s using it, he’s so on board. He sees how important it is and how much more comfortable he is. And so I think I have a good knowledge of the process. And I have a lot of aches and pains in my hips and my knees and so I tend to hold the railings all the time. I’m a total rail holder.”* –Female, age 61

This participant understood the risks of falling from watching her husband manage secondary osteoporosis and her mom manage primary osteoporosis. She knew the importance of taking additional precautions to avoid falling. Another participant has had to experience caregiving for her mother-in-law who had osteoporotic fractures, and her daughter who also experienced very low bone mineral density. Caregiving for others has made her more cautious about avoiding risks of fractures. She has modified her participation in certain activities out of a fear of fractures. She mentioned:

“*[my mother-in-law] would bang her leg against the toilet and break her leg. And our daughter … she just touches something. She constantly has a broken bone somewhere in her body, most of the time. Its’ really sad.” – Female, Age 88*

Due to the connection with her daughter’s high risk of fracture from being on medication for longer than recommended, this participant has gone against the advisement of her doctor to begin medication.

Few participants reported modifying their diet for their osteoporosis, there was a discussion on the fact that they already eat healthy and that should be sufficient for their osteoporosis as well. A couple of the participants sought advice from a dietitian to be able to meet their dietary goals and needs for osteoporosis, but most participants relied on the advice of their physicians for dietary advice.

### Awareness of environmental risks and opportunities

Environmental factors were more commonly described to affect the risk of falling for people with osteoporosis. No participants discussed environmental modifications to improve exercise or nutrition habits. Participants were able to identify that there are risks in their home environment and the outside environment, and often took measures to reduce the risk of falling. One participant recognized that carpets are a fall hazard and said “*we have taken our carpets”- Female, Age 88*. She described a situation where both her husband and her son tripped over the same rug in their hallway entrance. Seeing that the rug was a tripping hazard and increased their risk of falling she had it removed. She was quick to make home modifications to reduce the risk of falling and continue to live independently.

A common fear of falling in patients with osteoporosis is the Canadian winter conditions. One participant recently fell and explained her experience: *“I wasn’t walking! I just had a little patch of ice. And if I know it’s going to be icy then I put on the ice picks on my boots.”—Female, Age 60*

Although she recently fell, she does recognize that there were environmental modifications she could have made, by adding ice picks to her boots to reduce the risk of falling on ice. Other participants have mentioned improving their footwear choices to reduce the risk of falling during the winter season.

### Understanding the effect of exercise on the bones and in life

Interviews demonstrate that participants were confused of how much of a benefit exercise will have on the bones. One participant explained her internal conflict of whether she could manage her osteoporosis non-pharmacologically, because she did not want to begin pharmacological management. The quote below is an indication of her mental struggle to try to justify only using non-pharmacological intervention.

*“The logical part of my brain that what (exercise) it’s going to do is strengthen the muscles to support the bones so I’m more likely to um be stable and prevent myself from falling or recover if I slip. The magical thinking is (… …) there is a possibility of increasing bone density with exercise.”—*Female, age 57

Although the effect of the exercise on the bones remained confusing for some participants, the effect of the exercise in others life domains seems clear, even when the participant chooses to not do exercise, the knowledge about the benefit of exercising emerge.

“*At this point well, osteoporosis is a weakness of the bone so if you could keep the muscles and everything strong around the bones, it’s less likely you’ll fall and fracture it. I mean I know that. It’s not that I don’t. I just don’t do it*.” Female, Age 62

It was clear from the interviews that participants knew that exercise was beneficial but there seemed to be a lack of urgency to manage their osteoporosis because they couldn’t feel the disease. One participant explained “*I guess because it’s been a slow progress, I don’t notice it as much.”* Female, Age 62. This lack of urgency meant that they didn’t always adhere to the exercise recommendations or modify their lifestyle to reduce the risk of falls, or consider which nutritional needs they might need to better manage their osteoporosis.

### Challenges managing exercise expectations

A major concern for younger participants who were diagnosed with osteoporosis, was their possibility of return to pre-osteoporosis exercises. Specifically, one participant found that the advice provided to her from her physician, while conservative, might not have considered her own fitness levels. She said that the physician’s “*advice regarding exercise was very troublesome to me”* because the physician advised her to: “*stop cycling which is a very big part of my life. She said I can walk. And when I said what about yoga, she said there are all sorts of twists you can’t do.”;* the way in which this participant was told to manage her osteoporosis was very hard for the participant, since these activities were “*part of her identity”—*Female, age 57.

Another participant, who was very active prior to her diagnosis of osteoporosis, found that advice to stay active was very important to her. The participant made it clear that exercise is a very important part of her life.

*“I have been exercising pretty much all my life. More intensely since my mid 30´s. I think there are so many health benefits in terms of your cardiovascular health, you muscle development, bone strength, keeping at an optimal weight. But for me, it’s really keeping my mind at a healthy place. And releasing the good feel serotonin hormones. Making the stresses in your life a little more bearable, taking the time for yourself, to care for yourself, those kinds of things.” –Female, Age 51*

However, she also wanted to ensure that her participation in exercises were safe. She was worried that exercising might increase her risk of fracturing:

“*It’s having the barbell or dumbbell slip or evening tripping and falling over a dumbbell. So, I’m very cautious of where things are. But really it’s just being very careful about the movements and how they’re impacting your body.” Female, Age 51*

Conversely, one of the participants recognized the benefit of exercising with osteoporosis as: *“the confidence I have in walking. My balance, I mean even putting on my PJs.”—Female, Age 70.*

Participants consistently mentioned their frustration in not being able to fully return to their normal exercise routine, prior to their diagnosis with osteoporosis. One participant had a very complex medical history and expressed frustration when attempting to reach her fitness goals:

*“For every small goal you can achieve, it’s a large goal in the end. And when I first started the exercise program, I think I was frustrated with myself because I realized there were things, I couldn’t do but I kept thinking no you have to do them. Motivating yourself to continue to do them.” Female, Age 62.*

Her self-motivation allows her to continue to manage her osteoporosis through lifestyle modifications.

For some participants it was not about return to exercise, but rather being able to maintain daily activities. For one interviewee, she wanted to maintain her independence by continuing to participate in home maintenance and chores. She describes doing her daily activities as: “*I vacuum, I dust, I wash floors, I paint walls, I clean windows*”.—Female, Age 88. Maintaining daily activities was made possible for one of the participants by adapting a walking stick and making sure home modifications were installed, so he could continue grocery shopping and moving around in the neighbourhood.

Although fear has not stopped all participants from limiting their daily activities, one participant mentioned that she previously enjoyed hiking, but due to her concerns of falling, she has had to stop: *“It limits the hiking these days. I’m quite nervous about going down hills down. I always never minded going up, but I never liked going down, but now I’m actually terrified of going down”- Female, Age 59.*

Conversely, one participant mentioned that he does not prefer to be physically active, but has recognized the importance of exercise, since being diagnosed with osteoporosis. His preferred pre-diagnosis activity would be to sit down and read a book: *“I’d rather sit there with a good book. If I don’t force myself I won’t [be active]”—Male, Age 90.*

One participant mentioned that she prefers to stay active and does not let her osteoporosis play a factor when choosing activities. She mentioned that she took up golf and curling to connect with her husband and did not consider her osteoporosis when making those decisions.

*“I just try to keep doing what I’ve always done. Try to get there a couple times a week to get there for muscle bearing muscle class and try to work in as much cardio as I can. I like to count my steps if we’re walking. I curl in the winter so sometimes that interferes with the times I can go to the gym depending on how busy that is. And golf season starts, and I golf a little bit. And the gym has been a constant thing in my life. But curling and golf are a new thing for me, like in the last 5 years.” –Female, Age 59*

Other participants mentioned that their activity and nutrition is guided more by their personal preferences than their osteoporosis. One participant explained that despite her osteoporosis, she wishes to continue to ride her bike at the cottage and go on hikes. She was interested in learning about how to make those activities safer for her osteoporosis.

### Attitude towards non-pharmacological management

When observing participants discuss about their willingness to participant in an exercise programme, their attitude towards exercise contributed to their commitment to participating in exercise. Some of the participants felt that exercise was an integral part of their life and did not want to stop exercise for any reason. For those participants, the diagnosis of osteoporosis was challenging out of fear of having to stop exercising.

On the other side, there were participants who never enjoyed exercising and did not consider exercise as part of their identity: *“I have never really been an exercise person, that’s my fault. So, what I did at that point was I needed to up my vitamins and do other things. I knew I had a weakness which as exercise”.—Female, Age 62*

For these participants, being told that exercise is an important part of managing osteoporosis was difficult to hear, because they did not want to adhere to that portion of the recommendations. It seemed that the participants less engaged in exercise were much more aware of their nutrition and falls prevention needs and would manage their osteoporosis through their diet or using supplements.

Despite how aware some people were of the osteoporosis management recommendations or how well the information has been disseminated to them, a barrier to being adherent was other responsibilities and commitments. Largely, caregiving responsibilities contributed to not being able to exercise as often or in the same way the person would like to. For example, one participant mentioned she always enjoyed going on hikes with her husband, but recently his health had deteriorated, and she cannot go on the hikes anymore:

“*With [my caregiving role] I cannot swim or go to yoga or whatever. The different times with [her husband] have been when I put on the most weight. So, when he broke his pelvis and I was in the hospital every single day all day, and I really didn’t exercise, and I think I was comfort eating with lots of bad food.”* – *Female, Age 61*

As well, the participant mentioned that her nutrition is worse when her husband is sick, engaging in more alcohol consumption.

On the other side, one participant mentioned that she is more cautious about staying active and reducing the risk of falls because her husband has osteoporosis. She mentioned that although she also has osteoporosis, she is more concerned about caring for him: *“I think about his (risk of fracture), but I don’t’ think about mine … I’m more worried because he broke two arms last year.”—Female, Age 88*

## Discussion

This study aimed to better understand people with osteoporosis’ perspective on non-pharmacological management of osteoporosis and their perspective on acquiring knowledge about the disease. The following themes emerged: understanding fragility fractures and fall risk, understanding the effect of exercise on the bones and in life, challenges managing exercise expectations, knowledge acquisition through personal and vicarious experiences over the lifespan, awareness of environmental risks and opportunities and attitude towards having osteoporosis. Previous studies have identified barriers and facilitators to exercise in people with osteoporosis (Rodrigues et al., 2017; Ziebart, MacDermid et al., 2020; Ziebart et al., 2018), but this study advances the knowledge by discussing the exercise recommendations as well as nutrition and falls prevention strategies for managing osteoporosis.

The current study found that their attitude towards exercise dictated their willingness to participate in physical activity. Interestingly, a systematic review identifying the barriers and facilitators in participating in healthy behaviours found that participants with weight loss goals engaged in less physical activity than participants exercising for stress-relief and gaining a sense of wellbeing (Kelly et al., 2016). The current study corroborated these results in that the participants who exercised for their mental health or to feel good seemed more committed to returning to their pre-osteoporosis fitness. Another qualitative study occurring the UK similarly found that facilitators to engaging in exercise for people with osteoporosis were having clear tangible benefits and integrating the exercise into daily activities (Simmonds et al., 2016). Barriers were damaging joints, falling or other safety concerns, and conceptualizing the bones (Simmonds et al., 2016). It is important to understand that similar themes for barriers and facilitators of exercise are seen globally, and behaviour change interventions may benefit people with osteoporosis worldwide.

In the current study we found that participants were more likely to engage in non-pharmacological strategies if the participants were concerned about osteoporosis, as a disease and believed non-pharmacological intervention might help. Conversely many participants expressed not being able to feel the decline in bone mineral density and would forget that they had osteoporosis. In a qualitative study inquiring about people’s decision to take osteoporosis medication, they were less likely to take the medication because they did not believe that osteoporosis was a serious health concern (Sale et al., 2011), and were more likely to begin taking osteoporosis medication if they had trust in their healthcare provider (Sale et al., 2011).

One target population that may require additional attention is men with osteoporosis. The current study only had one male participant, limiting the ability to understand osteoporosis management from the male’s perspective. Other studies have explored the male’s perspective and found that men were not as well aware of their osteoporosis or how to manage it (Bombak and Hanson, 2016). A systematic review was conducted specifically to understand men’s perception of living with osteoporosis, identified four publications, indicating that there is a substantial research gap (Compton et al., 2019). The review concluded that there is a gap in the healthcare delivery supports for osteoporosis that preferentially disadvantages men (Compton et al., 2019), such as men not being flagged in the emergency room as at risk for osteoporosis as often as women. When it comes to engaging in activity, it was found that men were more risk-takers as they did not want to limit their lifestyle, and minimized the importance of their diagnosis (Compton et al., 2019; Ziebart et al., 2018). In the current study, the one male participant said he relied heavily on his wife for his food, home modifications, appointments, and recommendations on how to manage his osteoporosis. Relying on the support of a spouse or family member might be one strategy to improve the update of osteoporosis recommendations for men.

Conceptualizing this complex topic may be further facilitated through the International Classification of Functioning, Disability and Health (ICF) framework. The ICF framework is helpful to account for not only the disease (osteoporosis) and the body function (a fracture or declining bone mineral density) but also the participant’s activity, participation, environmental factors, and personal factors. The ICF has been used as a framework for people with osteoporosis (Ziebart, Page et al., 2020) and could also be applied to this topic both through research and for clinical management.

Clinically, there are several implications that can be drawn from the results of this study. It seemed that many of the participants were interested in non-pharmacological management of osteoporosis, but weren’t always clear how to engage in exercise, and falls prevention strategies. In this case, patients should be seeking out, and physicians should be referring patients to a BoneFit™ trained physiotherapist. Not all physiotherapists are trained in nutrition, so seeking support from a dietitian would also be beneficial. People with osteoporosis should be encouraged to remain active, but special considerations should be made towards how the activity is being done, which activities are prioritized, and proper alignment should always be encouraged (Giangregorio et al., 2014).

This study uniquely asked participants about exercise, nutrition, and falls prevention strategies for treating osteoporosis, rather than focusing on only one treatment strategy, providing insight into treatment management for people with osteoporosis. A variety of patients with osteoporosis were interviewed with an age range of 51–90, from a variety of ethnical backgrounds and from both rural and urban cities. There are several limitations in this study. Firstly, it is acknowledged by the author that there may be personal bias associated with the analysis and questions, the methodology was selected to account for that, and to provide a more clinical perspective to the data. Further, the participants were eager to participate in the study and may have been more proactive with their health care professionals to seek information, potentially leaving themes unknown for patients that are passive with their osteoporosis care. Participants were recruited from one geographical location, decreasing the generalizability of these results. Finally, some of the interviews took place in a public location, with consent from the participant, but it is possible that those participants may not have spoken as freely.

In conclusion, this study focused on the non-pharmacological care for people with osteoporosis. Participants recognized the benefit of non-pharmacological management for managing osteoporosis, but sometimes found it difficult to integrate into their daily activities due to lack of time or knowledge. Participants weren’t always clear on which component of their osteoporosis management should be prioritized. More research is required to address men with osteoporosis, and behaviour change strategies to better ensure people with osteoporosis are adhering to the non-pharmacological osteoporosis recommendations are required.
